# Impact of mesenchymal stem cell size and adhesion modulation on in vivo distribution: insights from quantitative PET imaging

**DOI:** 10.1186/s13287-024-04078-4

**Published:** 2024-11-28

**Authors:** Xin Ji, Lizhen Wang, Yudan Zhong, Qian Xu, Junjie Yan, Donghui Pan, Yuping Xu, Chongyang Chen, Jing Wang, Guangji Wang, Min Yang, Tiannv Li, Lijun Tang, Xinyu Wang

**Affiliations:** 1 Department of Nuclear Medicine, The First Affiliated Hospital of Nanjing Medical University, Jiangsu Province Hospital, Nanjing, 210029 P.R. China; 2 Department of Radiopharmaceuticals, School of Pharmacy, Nanjing Medical University, Nanjing, 211166 P.R. China; 3 NHC Key Laboratory of Nuclear Medicine, Jiangsu Key Laboratory of Molecular Nuclear Medicine, Jiangsu Institute of Nuclear Medicine, Wuxi, 214063 P.R. China; 4 Jiangsu Renocell Biotech Co., Ltd., Nanjing, 211100 P.R. China; 5 Key Laboratory of Drug Metabolism and Pharmacokinetics, State Key Laboratory of Natural Medicines, China Pharmaceutical University, Nanjing, 211198 P.R. China

**Keywords:** Mesenchymal stem cell, Adhesion capacity, Cell size, 3D spheroid, Positron emission tomography

## Abstract

**Background:**

Successful engraftment and localization of mesenchymal stem cells (MSCs) within target tissues are critical factors influencing their therapeutic efficacy for tissue repair and regeneration. However, the relative contributions of biophysical factors like cell size and adhesion capacity in regulating MSC distribution in vivo remain incompletely understood.

**Methods:**

Cell adhesion peptides and hanging drop method were used to modify the adhesive capacity and size of MSCs. To quantitatively track the real-time biodistribution of transplanted MSCs with defined size and adhesion profiles in living mice and rats, the non-invasive positron emission tomography (PET) imaging was applied.

**Results:**

Surface modification with integrin binding peptides like RGD, GFOGER, and HAVDI reduced MSC adhesion capacity in vitro by up to 43.5% without altering cell size, but did not significantly decrease lung entrapment in vivo. In contrast, culturing MSCs as 3D spheroids for 48 h reduced their cell diameter by 34.6% and markedly enhanced their ability to pass through the lungs and migrate to other organs like the liver after intravenous administration. This size-dependent effect on MSC distribution was more pronounced in rats compared to mice, likely due to differences in pulmonary microvessel diameters between species.

**Conclusion:**

Our findings reveal that cell size is a predominant biophysical regulator of MSC localization in vivo compared to adhesion capacity, providing crucial insights to guide optimization of MSC delivery strategies for enhanced therapeutic efficacy.

**Graphical abstract:**

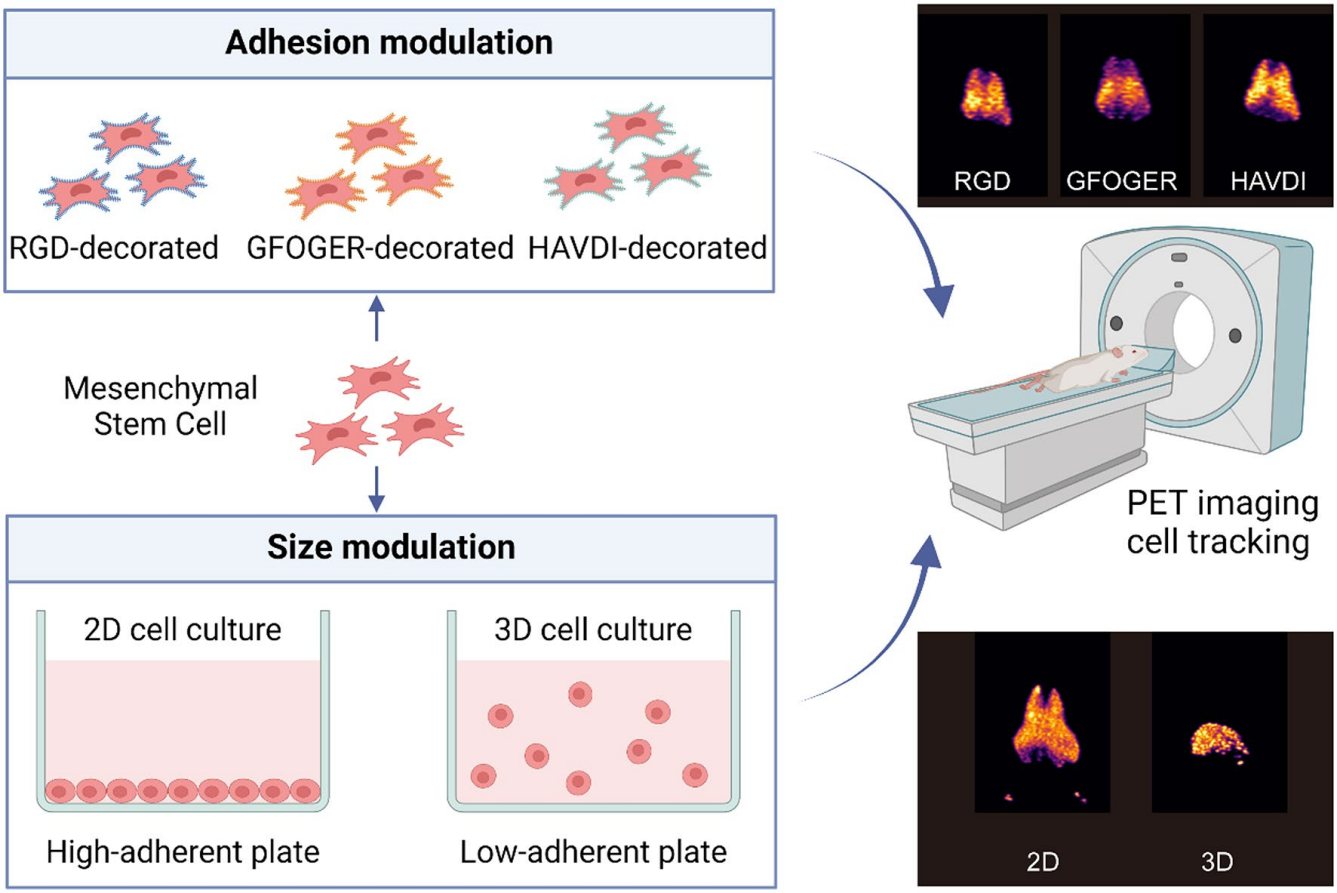

**Supplementary Information:**

The online version contains supplementary material available at 10.1186/s13287-024-04078-4.

## Introduction

Mesenchymal stem cells (MSCs) represent a beacon of hope in the realm of tissue repair and regeneration, driven by their remarkable multipotent differentiation capacity and proficient paracrine signaling abilities [1, 2]. Human Umbilical cord blood-derived MSCs (hUC-MSCs) have garnered increasing attention in clinical applications owing to their easy accessibility, low immunogenicity, and lack of ethical concerns, especially in comparison to bone marrow-derived MSCs [3]. Within the landscape of regenerative medicine, the spatial distribution and successful engraftment of MSCs within target tissues emerge as pivotal factors, profoundly influencing their therapeutic efficacy [4–6]. Despite their immense potential, a substantial hurdle persists: the need to meticulously orchestrate the in vivo localization and sustained retention of administered MSCs to unlock their full reparative prowess [7]. Addressing this challenge is imperative to harnessing the transformative potential of MSC-based therapies, ensuring optimized tissue regeneration and ultimately, improved clinical outcomes for patients grappling with tissue damage or disease.

Prior studies have investigated various factors that may influence MSC distribution and homing, including cell surface adhesion molecules [8–10], chemokine gradients [11], and mechanical properties of the microenvironment [12]. In particular, the role of cell size and adhesion capacity in regulating MSC dispersal and homing has been an area of active research [13]. Some reports have suggested that MSCs with greater adhesive interactions may be more effectively retained within target tissues [10, 14]. However, other work has indicated that smaller MSCs may be able to better extravasate and permeate into tissues [13]. However, the relative contributions of cell size versus adhesion in governing in vivo MSC distribution remain incompletely understood.

In recent years, positron emission tomography (PET) imaging has emerged as a powerful non-invasive technique to track the real-time biodistribution of transplanted cells within living subjects, in both the preclinical realm of foundational research and the clinical arena of real-world application [15]. PET offers high sensitivity and quantitative capabilities for monitoring the trafficking and engraftment of cell-based therapies, including MSCs, within target organs [16, 17]. ^89^Zr is an advantageous PET isotope for longitudinal tracking compared to ^18^F, ^64^Cu, owing to its half-life of 78.4 h and low radiation exposure. ^89^Zr-oxinate [18] and ^89^Zr-DBN(p-NCS-Bz-DFO) [19] are the two most commonly applied labeling methods. ^89^Zr-oxine labeling relies on passive diffusion, which can achieve high cell labeling efficiency with simple labeling procedure. But this methodology exhibits significant efflux during metabolism and cell death compared to ^89^Zr-DBN, making it difficult to distinguish between living cells, cell debris, or leaking radionuclides. Recently, our research group has achieved a significant leap in enhancing the precision of PET tracer cells through the innovative removal of free radionuclides from the biological milieu [20]. This advanced imaging modality has been increasingly utilized to provide critical insights into the factors regulating in vivo cell localization and retention, spanning from pre-clinical research to clinical application [21, 22].

The current study aims to directly address this knowledge gap by leveraging non-invasive PET imaging to track the real-time biodistribution of hUC-MSCs with defined size and adhesion profiles. Through this innovative imaging approach, we seek to elucidate the predominant biophysical regulator of mesenchymal stem cell localization within host tissues. Findings from this work will provide crucial insights to guide the design of MSC-based therapies and enhance their therapeutic potential for tissue repair and regeneration.

## Materials and methods

### Materials

Agents including 8-Hydroxyquinoline (oxine), Na_2_CO_3_, and 4-(2-hydroxyethyl)-1-piperazineethanesulfonic acid (HEPES) solution were procured from Sigma-Aldrich (St. Louis, MO). Dimethyl sulphoxide (DMSO) was obtained from Acros Organics (Belgium, USA). ^89^Zr-oxalate was supplied by Dongcheng AMS Pharmaceutical Co., Ltd (Nanjing, China). β-glycerol phosphate, ascorbic acid, 3-isobutyl-1-methylxanthine, indomethacin and Alizarin red S (ARS) were obtained from Innochem Science & Technology Co., Ltd. (Beijing, China). Dexamethasone was obtained from MedChemExpress (New Jersey, USA). GAPDH internal reference primers, along with integrin primer sequences for real-time qPCR were obtained from Sangon Biotech Co., Ltd. (Shanghai, China). Peptides including RGD (arginine-glycine-aspartic acid), GFOGER (glycine-phenylalanine-hydroxyproline-glycine-glutamic acid-arginine), HAVDI (histidine-alanine-valine-aspartic acid-isoleucine) and fluorescence-conjugated peptide including RGD-FITC (fluorescein 5-isothiocyanate), GFOGER-FITC, HAVDI-FITC were all obtained from Sangon Biotech Co., Ltd. (Shanghai, China). Fibronectin and collagen (Type I, from rat tail) was obtained from Yeasen Biotech Co., Ltd. (Shanghai, China)

### Cell culture and animals

The hUC-MSCs were directly obtained from cell injections (National Medical Products Administration of China approved for lupus nephritis) provided by Jiangsu Ruiyuan Biotechnology Co., Ltd (Jiangsu, China) [23]. All cells were cultured in DMEM/F12 (Gibco) medium supplemented with 10% fetal bovine serum (Gibco) and 1% penicillin − streptomycin (Beyotime Biotechnology, Shanghai, China). The culture was maintained at 37 °C in a humidified atmosphere containing 5% CO_2_.

For three dimensional (3D) culture, passage 6 hUC-MSCs were cultured using hanging drop method. Twenty thousand MSCs in 25 µl medium per drop were seeded in hanging drops and incubated for 0–72 h to form 3D spheroids. To obtain single cells, spheroids were incubated with accutase for 10–20 min (according to the 3D culture time) with gentle pipetting every 2–3 min.

All animal experiments were conducted in accordance with the National Institutes of Health guidelines for the care and use of laboratory animals and were approved by the Institutional Animal Care and Ethics Committee of Jiangsu Institute of Nuclear Medicine (Wuxi, China) (approval number: JSINM-2023-100). Adult female BALB/c mice (6–8 weeks old, *n* = 30) and Wistar rat (150–200 g, *n* = 10) were purchased from Vital River (Zhejiang, China). All animals were maintained under specific-pathogen-free (SPF) condition with a constant temperature (23 ± 1.5 °C) and humidity (70 ± 20%) on a 12-hour light/dark cycle. Animals were anesthetized with 2% isoflurane in oxygen during each scan and remained awake between imaging sessions. At the end of the study, euthanasia was confirmed by cervical dislocation. The work has been reported in line with the ARRIVE guidelines 2.0.

### Cell adhesion assay

Cell adhesion assays were performed in 24-well plates that were coated with fibronectin (FN) or collagen I (CI) at a concentration of 5 µg/cm^2^. Wells were then washed 3 times with PBS and blocked with 1% bovine serum albumin (BSA) in PBS for 1 h at 37 °C. 1% BSA in PBS alone-coated wells was used as a control. HUC-MSCs were preincubated with RGD, GFOGER and HAVDI at concentrations of 0, 0.01, 0.1, 1, 10 µg/ml for 15 min and suspended every 5 min. The hUC-MSCs were subsequently washed 3 times with PBS and resuspended with serum-free medium. Then 1 × 10^5^ hUC-MSCs were seeded into each well and incubated for 30 min at 37 °C. The number of adherent cells was calculated using CellTiter-Lumi kit (Beyotime Biotechnology, China). The experiment was performed in quadruplet wells for each variable.

### Flow cytometry

HUC-MSCs were suspended in PBS containing 1% BSA at 10^6^ per ml. 200 µL cell aliquots were incubated with fluorescence-conjugated peptide: RGD-FITC, GFOGER-FITC, HAVDI-FITC at concentrations of 5, 20, and 40 µg/ml for 15 min. Then the hUC-MSCs were washes 3 times with PBS. A total of 10,000 events were analyzed by flow cytometry (BD FACSCalibur, San Jose).

### Radiolabeling of hUC-MSCs

For constructing ^89^Zr-oxine, the pH of the ^89^Zr^4+^ solution was adjusted to 7 using 1 M Na_2_CO_3_ and 0.1 M HEPES buffer. Then, 37 MBq ^89^Zr^4+^ solution was mixed with 10 µg oxine (20 µg/µL dissolved in DMSO) at room temperature for 15 min. The formation of ^89^Zr-oxine complexes was confirmed through radio-TLC analysis [24], using 100% methanol as the mobile phase on ITLC-SG chromatography paper. To labeling hUC-MSCs, every 10^6^ cells were incubated with 0.37 MBq ^89^Zr-oxine solution at room temperature for 15 min (the prepared oxine solution was added into 1 ml 10^7^cells/ml cell PBS suspension with a final DMSO concentration of less than 0.2%) and then centrifuged at 300 ×G for 3 min. And the labeling dose of 0.37MBq/million cells with 80% ^89^Zr retention post-labeling has been identified in previous work that exhibits no adverse effect on cell viability, function, or phenotype [20]. Finally, the ^89^Zr-labeled hUC-MSCs were washed with PBS 3 times. The cell labeling efficiency was calculated based on the radioactivity of the cells and supernatant measured by radioactivity meter (PTW, Germany).

### Osteogenic differentiation

hUC-MSCs were seeded in 6-well plates at a density of 5 × 10^4^ cells per well and cultured in osteogenic medium consisting DMEM (Adamas) supplemented with 10% FBS, 1% penicillin-streptomycin, 10 mM β-glycerol phosphate, 50 µM ascorbic acid and 0.1 µM dexamethasone. The culture medium was replaced every 3 days, and ARS staining was used to detect bone matrix formation at day 14.

### Adipogenic differentiation

hUC-MSCs were seeded in 6-well plates at a density of 2 × 10^5^ cells per well and cultured with adipogenic differentiation medium consisting of DMEM (Adamas) supplemented with 10% FBS, 1% penicillin − streptomycin, 1 µM dexamethasone, 0.5 mM 3-isobutyl-1-methylxanthine and 50 µM indomethacin. The medium was replaced every 3 days, and Oil Red O staining was used to detect the potential of adipogenic differentiation on day 21.

### Quantitative polymerase chain reaction (qPCR)

Real-time PCR was used to quantify the expression of integrin subunits in two dimensional (2D) and 3D hUC-MSCs. hUC-MSCs were harvested 24–48 h after 2D or 3D culture. PCR assays were performed by Applied Biosystems ABI 7500 system (Thermo Fisher, USA). Reactions were performed in 20 µL containing 10 µL of qPCR SYBR Green mix (Yeasen Biotech, China), 0.4 µL of forward and reverse primers, and 20 ng of template gDNA diluted in water. The SYBR Green PCR reaction conditions were as follows: the holding phase was 95 °C for 2 min. Cycles were 40 cycles of 95 °C for 10 s and 60 °C for 34 s. All PCR assays were performed in triplicate. Primer sequences were shown in Table S1.

### Histological analysis

The lungs and kidneys from sacrificed mice and rats were dissected into small pieces of approximately 3 cm^3^. Five representative pieces of tissues for each organ at different sites were fixed with 4% paraformaldehyde for 48 h. Fixed tissues were subjected to water cleaning, dehydration, and paraffin embedding. Paraffinized tissues were sectioned to 5 μm thick slices.

For histochemical staining, deparaffinized tissue sections were stained with hematoxylin and eosin (H&E) to evaluate the morphology of the kidney. The stained sections were scanned and photographed using Olympus IX51 Microscopy.

For immunofluorescence staining, each section was incubated with α-smooth muscle actin (α-SMA) antibody (Proteintech, 14395-1-AP) at a dilution of 1:500. The nuclei were counterstained with 4,6-diamidino-2-phenylin-dole (DAPI) for 10 min. The stained sections were photographed using Olympus IX51 Microscopy. For measuring the diameter of lung microvasculature, only structures lined with α-SMA^+^ cells and filled with red blood cells were measured as arterioles or venules. Around 20 arterioles or venules were measured on each section for statistical analysis.

### In vivo micro-PET imaging and data processing

PET scans were performed on an Inveon microPET scanner (Siemens Solutions, Germany). BALB/c mice and Wistar rats were intravenously injected with 6 × 10^5^ and 1.2 × 10^6 89^Zr-radiolabeled hUC-MSCs via the tail vein, respectively. The radioactivity of each mouse was 111–148 KBq and rat was 259–333 KBq. And 10 min-long static PET scans were performed at 30 min, 2, 4, 6, 24, and 48 h post injection. The conduct of the injection and group allocation were all blinded. To remove free radionuclides from the biological milieu, 10 mg/ml DFO (Deferoxamine mesylate, Novartis China) was administered intramuscularly 30 min before hUC-MSCs injection at a dosage of 50 mg/kg per mouse and three times per day thereafter. The image data was further analyzed using ASIPro software. The percentage injected dose per gram or milliliter (%ID/g or %ID/ml) of tissue was calculated based on regions of interest (ROIs) that manually delineated. Besides, the maximum intensity projection (MIP) figures based on PET scans was generated using PMOD Fusion Tool.

### Ex vivo biodistribution

After the last imaging time point, heart, liver, spleen, lung, kidney, gastric, small intestine, muscle, joint, femur were harvested and weighed for biodistribution analysis using a gamma counter (Wizard 2480, PerkinElmer, Waltham, MA).

### Statistics

GraphPad Prism 9.0 software was used to perform statistical analysis. The data were presented as mean ± SD, unless otherwise noted. The statistical significance was evaluated using one-way ANOVA or student t test according to homogeneity of variance: *, *p* < 0.05; **, *p* < 0.01; ***, *p* < 0.001; ****, *p* < 0.0001; ns, not significant, *p* > 0.05.

## Results

### Cell surface modification alters cell adhesive capacity

**Fig. 1:**
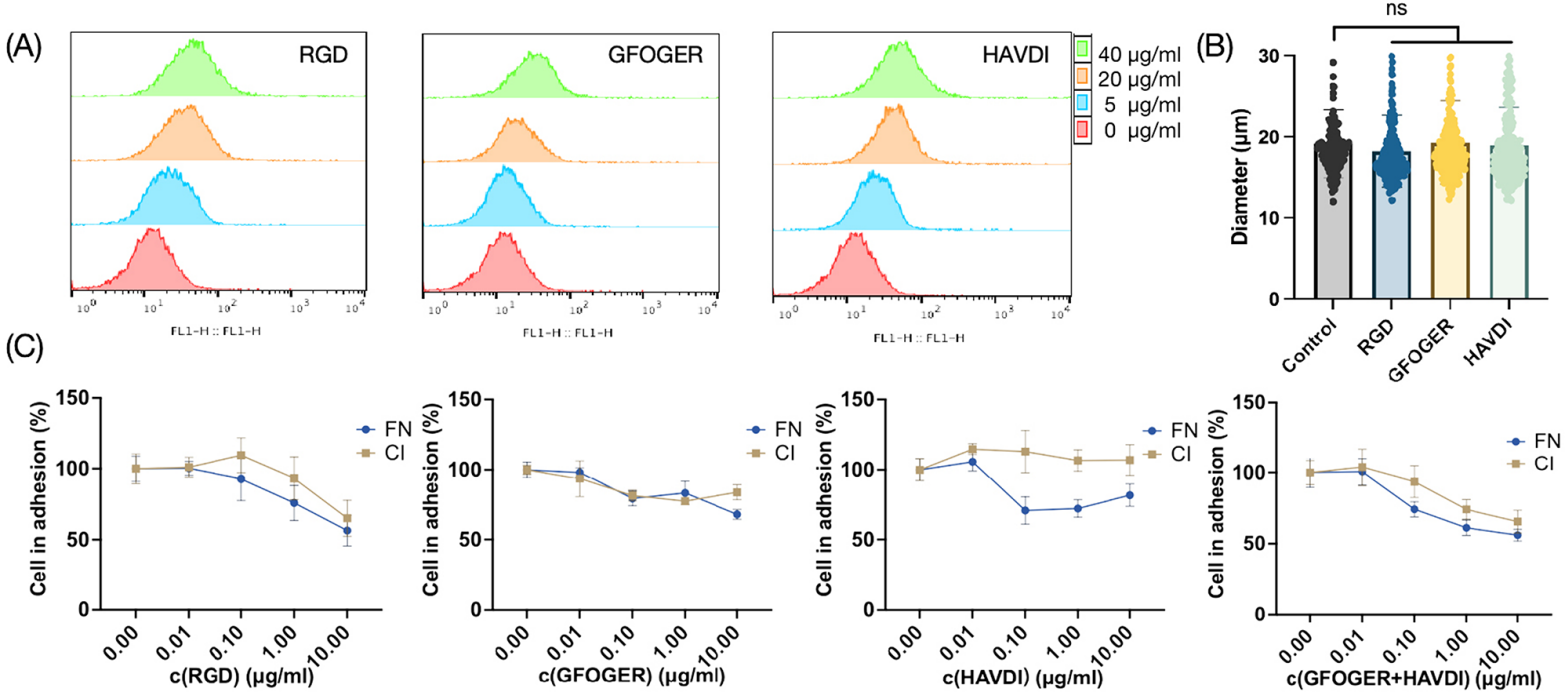
In vitro evidence demonstrates cell surface modification alters cell adhesive capacity. (**A**) Flow cytometry assessment of hUC-MSCs binding affinity after incubation with different concentration of RGD-FITC, GFOGER-FITC, and HAVDI-FITC. (**B**) The diameter was assessed after hUC-MSCs incubated with various peptides. (**C**) Peptide-preincubated hUC-MSCs were seeded on fibronectin (FN) or collagen I (CI) monolayers and incubated for 30 min, adherent cells were then calculated (*n* = 4). Values are expressed as the means ± SD. ns, not significant, *p* > 0.05

Integrins are expressed on the cell surface and bind to extracellular matrix (ECM) adhesion proteins such as fibronectin and collagen I, playing an important role in cell adhesion. RGD (a fibronectin mimic) and GFOGER (a collagen mimic) derived from ECM adhesion protein and and HAVDI (an N-cadherin mimic) from cell-cell interaction proteins were applied to transiently decorate cell surface, thus reducing cell adhesion capacity with minimally impacts on their secretory profiles [25–27]. As depicted in Fig. 1A, the integrin binding peptides bound to the hUC-MSC surface were proportional to the peptide concentration. Notably, at the concentration of 10 µg/ml, RGD, GFOGER and HAVDI pre-treatment had no discernible impact on cell diameter, with the value of 18.23 ± 4. 47, 19.25 ± 5.15, 18.92 ± 4.68 compared to control group of 19.12 ± 4.23 μm (Fig. 1B). RGD, GFOGER and HAVDI pre-treatment decreased hUC-MSC attachment to the FN/CI monolayers to 56.5%/65.3%, 67.9%/83.7% and 81.7%/107.0%, respectively, and the combination of GFOGER and HAVDI could further decrease hUC-MSC attachment to 56.0%/65.5% (Fig. 1C).

### Radiolabelling of hUC-MSCs with ^89^Zr

The radionuclide ^89^Zr was used to radiolabel hUC-MSCs through the ^89^Zr-oxine labeling method. The radiochemical yield of ^89^Zr-oxine was approximately 80%, as determined by Radio-iTLC, ensuring the high efficiency of cell radiolabeling (Figure S1). A previously reported safe radioactivity level was chosen to ensure that the radiolabeling process did not impact key stem cell properties, such as stemness or senescence [20]. The cell labeling efficiency for 2D cultured hUC-MSCs was about 58.4-75%, for 3D cultured hUC-MSCs were 40-48.4% (Figure S2). And the final retained dose per cell were 222-277.5 kBq/ million cells for 2D hUC-MSCs and 222-244.2 kBq/million cells. At this safe dosage, cell viability is not affected. The survival rate of ^89^Zr-labeled hUC-MSCs remained consistent with that of unlabeled hUC-MSCs, both exceeding 95% within three days post-labeling (Figure S3). Furthermore, the retention of ^89^Zr in radiolabeled hUC-MSCs was assessed over a 5-day period following initial radiolabeling. The results showed that approximately 80% of the ^89^Zr retained in the cells over this duration, indicating stable labeling and minimal loss of the radionuclide during the observation period (Figure S4).

### PET tracking of surface-modified hUC-MSCs distribution in BALB/c mouse

**Fig. 2:**
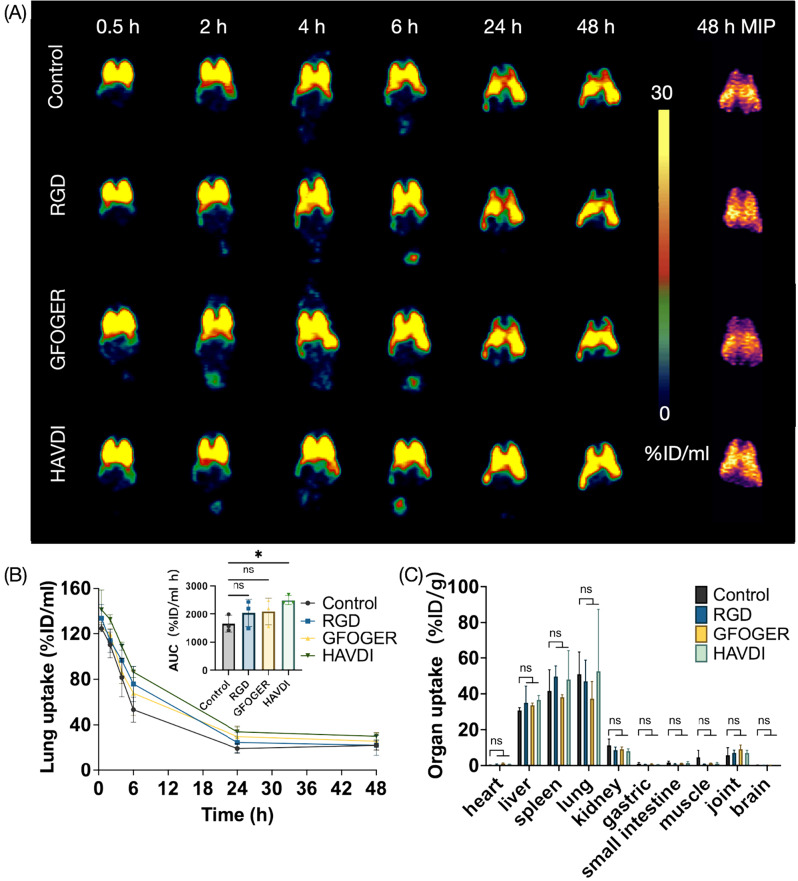
Effects of cell adhesion capacity on hUC-MSCs distribution in vivo. (**A**) Representative PET images of BALB/c mice injected with ^89^Zr-labeled hUC-MSCs at various time point within 48 h. The hUC-MSCs were pre-incubated with RGD, GFOGER, HAVDI and PBS as a control before radiolabeling. (**B**) Lung uptake of the ^89^Zr-labeled hUC-MSCs based on the PET imaging (*n* = 3). (**C**) Ex vivo biodistribution of intravenously transplanted ^89^Zr-labeled MSCs in BALB/c mice (*n* = 3). Values are expressed as the means ± SD. *, *p* < 0.05; ns, not significant, *p* > 0.05

In order to investigate the impact of adhesion capacity on hUC-MSCs distribution in vivo, we utilized PET to track ^89^Zr-radiolabeled surface-modified hUC-MSCs. The coronal PET images captured over a 48-hour period indicated that the initial distribution of the hUC-MSC transplants is predominantly in the lungs, with subsequent migration observed in organs such as the livers and spleens (Fig. 2A). This trend is similar among the groups with and without peptide-decoration. Lung uptake was analyzed based on the PET image (Fig. 2B), revealing that peptide pre-treatment did not decrease the entrapment of MSCs in the lungs, and in fact, showed an increase when treated with HAVDI (*p* = 0.044). To comprehensively characterize the biodistribution of MSCs, mice were further sacrificed at 48 h post-injection for radioactivity measurement of various organ (Fig. 2C). And the distribution of radioactivity was found to be similar among four groups.

### 3D culture reduces the volume of hUC-MSCs and remains pluripotency

**Fig. 3:**
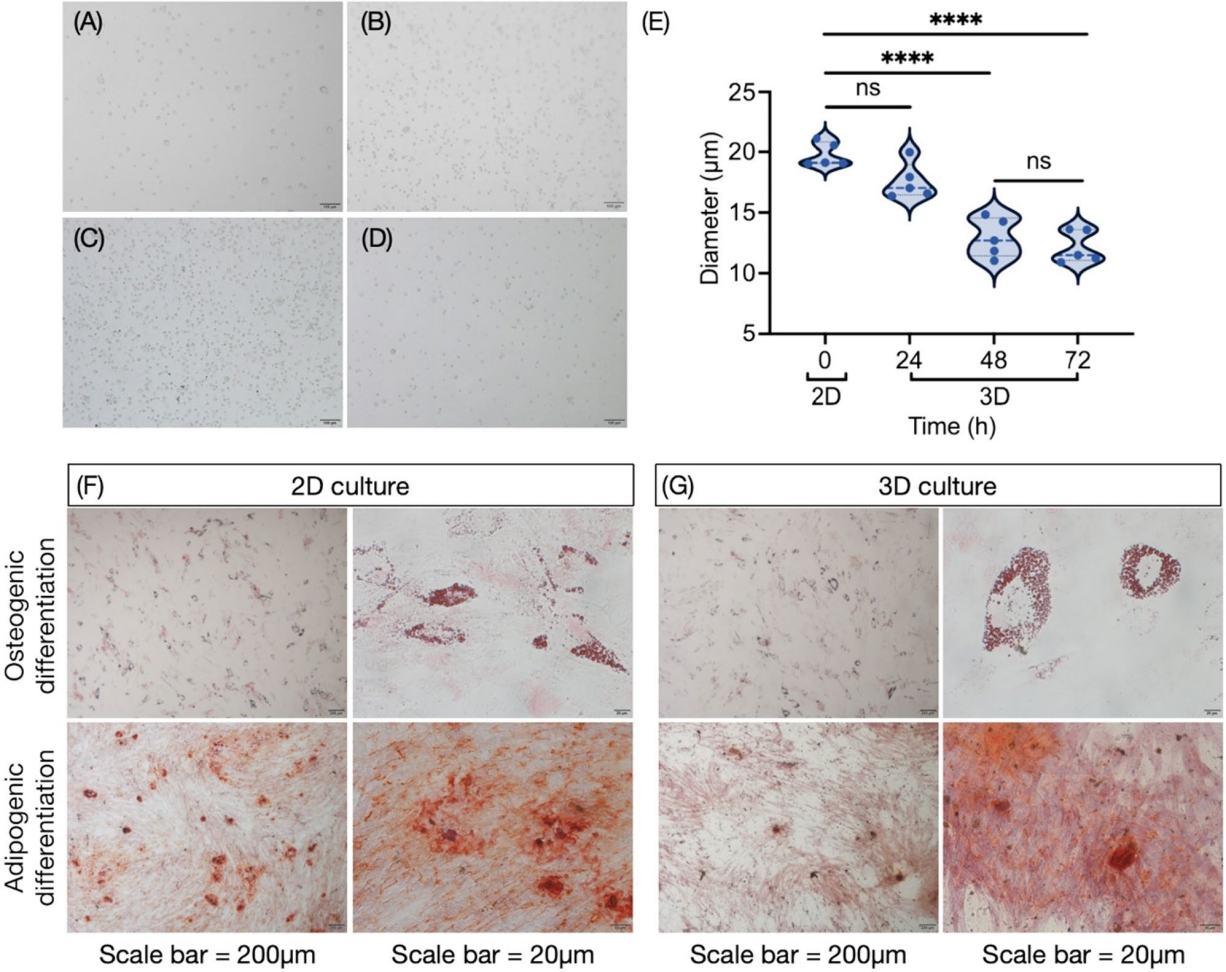
3D culture reduces cell volume. Representative microscopic images of hUC-MSCs cultured in (**A**) 2D, (**B**) 3D for 24 h, (**C**) 3D for 48 h, and (**D**) 3D for 72 h, scale bar = 100 μm. (**E**) The diameters of hUC-MSCs under various culture environment were calculated using ImageJ. (**F**) The differentiation ability of 2D-cultured hUC-MSCs. Alizarin red staining shows calcium content and Oil Red O staining shows lipid droplets in cells. (**G**) The lineage differentiation potential of 3D-cultured hUC-MSCs. The higher resolution figure are available in the supplementary materials. *, *p* < 0.05; **, *p* < 0.01; ***, *p* < 0.001; ****, *p* < 0.0001; ns, not significant, *p* > 0.05

Hanging drop method was applied to form 3D hUC-MSC spheroid. To investigate the influence of 3D incubation on hUC-MSCs, cells dissociated from 2D monolayer (0 h) and 3D-cultured spheroids at 24, 48 and 72 h were collected (Fig. 3A,B,C,D, Figure S5-S8). As depicted in Fig. 3E, the diameter of cells dissociated from spheroids cultures 48 h (12.93 ± 1.60 μm) and 72 h (12.17 ± 1.33 μm) were significantly smaller than those from 2D monolayers (19.78 ± 0.99 μm), while 24-hour 3D culture did not affect diameter. Therefore, a hanging drop culture period of 48 h appears to be an optimal condition for obtaining 3D hUC-MSCs. Furthermore, we constructed lineage differentiation potential assays to determine hUC-MSC differentiation potential. Differentiated osteocytes and chondrocytes were observed in both 2D and 3D cultured hUC-MSCs (Fig. 3F and G, Figure S9-S10). Additionally, we investigated the impact of 3D culture on the senescence of hUC-MSCs. Our findings indicate that 3D culture conditions did not promote or exacerbate cellular senescence, suggesting that this culture method preserves the youthful phenotype of hUC-MSCs (Figure S11).

**Fig. 4:**
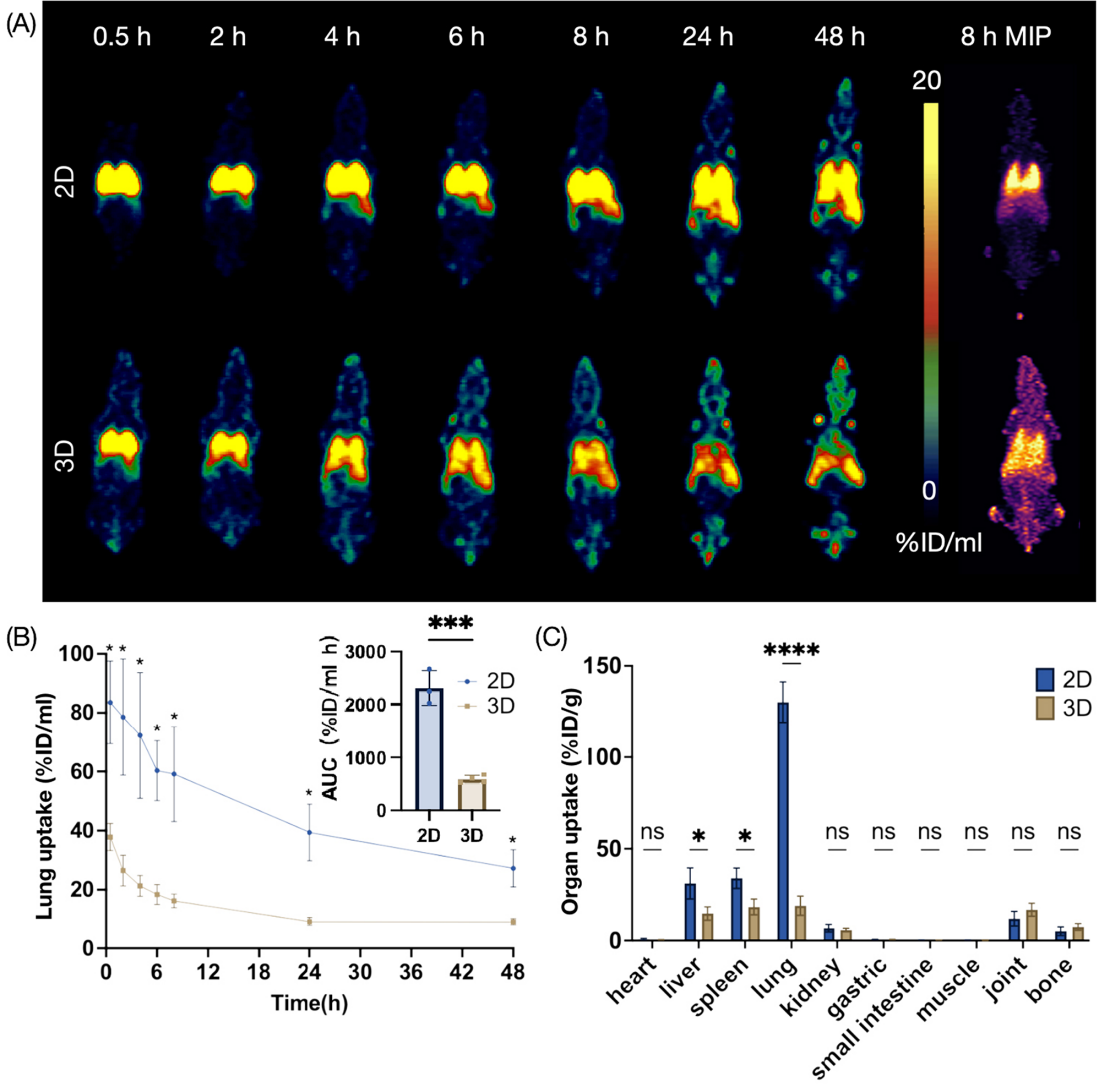
Effects of cell size on hUC-MSCs distribution in BALB/c mice. (**A**) Representative PET images of BALB/c mice injected with ^89^Zr-labeled hUC-MSCs derived from 2D monolayers and 3D spheroids at various time point within 48 h. (**B**) Lung uptake of the ^89^Zr-labeled hUC-MSCs based on the PET imaging (*n* = 4). (**C**) Ex vivo biodistribution of intravenously transplanted ^89^Zr-labeled hUC-MSCs in BALB/c mice (*n* = 4). Values are expressed as the means ± SD. *, *p* < 0.05; **, *p* < 0.01; ***, *p* < 0.001; ****, *p* < 0.0001; ns, not significant, *p* > 0.05

**Fig. 5:**
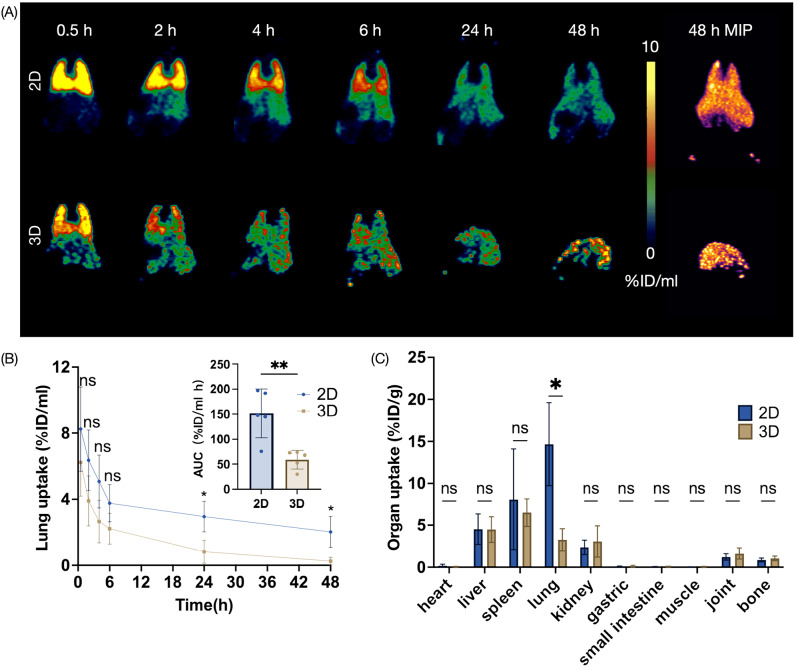
Effects of cell size on hUC-MSCs distribution in Wistar rat model. (**A**) Representative PET images of Wistar mice injected with ^89^Zr-labeled hUC-MSCs derived from 2D monolayers and 3D spheroids within 48 h. (**B**) Lung uptake of the ^89^Zr-labeled hUC-MSCs based on the PET imaging (*n* = 5). (**C**) Ex vivo biodistribution of intravenously transplanted ^89^Zr-labeled hUC-MSCs in Wistar mice (*n* = 5). Values are expressed as the means ± SD. *, *p* < 0.05; **, *p* < 0.01; ***, *p* < 0.001; ****, *p* < 0.0001; ns, not significant, *p* > 0.05

Furthermore, we also evaluated the effects of 3D culture on the expression of hUC-MSCs through real-time PCR analysis. 2D cultured hUC-MSCs expressed much lower levels of all integrins examined except for integrin α6 (Figure S12). Besides, prolonged 3D culture duration led to the increased expression of most integrin except for integrin α6.

### PET tracking of 3D-cultured hUC-MSCs distribution in BALB/c mouse

To investigate the impact of cell size on hUC-MSC distribution, we obtained the coronal PET images over a 48-hour period following intravenous administration of 3D hUC-MSCs dissociated from spheroids (3D group) and 2D hUC-MSCs dissociated from monolayers (2D group). As depicted in Fig. 4A, both group exhibited initial retention in lungs and subsequently migrated to other organs like liver and spleen. Of note, 3D group demonstrated reduced retention in the lungs and a more rapid migration of cells to livers and blood pool in comparison to 2D group. Lung uptake was analyzed based on the PET images, revealing a notable decrease in lung hUC-MSCs retention after 3D culture, while exerting no apparent impact on liver uptake (Fig. 4B and Figure S13A). Ex vivo biodistribution were analyzed 48 h post-injection (Fig. 4C). Consistently, 3D group exhibited lower levels of radioactivity in the lungs when compared to the 2D group, with values of 18.99 ± 5.23 and 130.07 ± 11.13%ID/g, respectively. Also, uptake in the liver and spleen uptake were significantly reduced in 3D group.

We evaluated the retention of hUC-MSCs in the lungs by injecting DiO-labeled cells into BALB/c mice and analyzing tissue sections. The results showed that a greater number of 2D-cultured hUC-MSCs remained in the lung tissue compared to 3D-cultured hUC-MSCs (Figure S14), indicating distinct distribution patterns between the two culture conditions. Additionally, based on the observed size of the green fluorescent clusters, these signals appear to represent clusters of cells rather than individual cells. Notably, the short-term retention of these cells did not cause any notable pathological changes in the tissues, aside from a transient phase of mild pulmonary edema (Figure S15).

### PET tracking of 3D-cultured hUC-MSCs distribution in Wistar rats

We further applied Wistar rats to evaluate the distribution of 3D hUC-MSCs in different models (Fig. 5A). The results showed that 3D hUC-MSCs rapidly passed through the lungs and exhibited relatively low level of uptake since 4 h post-injection. Based on PET images, we observed a notable reduction in lung uptake in the 3D group compared to the 2D group at 24 h and 48 h, with values of 0.82 ± 0.70 vs. 2.96 ± 0.93%ID/g and 0.25 ± 0.23 vs. 2.03 ± 0.94%ID/g, respectively (Fig. 5B). Also the area under the curve (AUC) of lung uptake in the 3D group was significantly lower than that in the 2D group (*p* = 0.004), indicating lower hUC-MSC retention and lower absorbed doses in the lungs. Although the liver uptake in the 3D group was relatively higher at the 0.5-hour time point, there was no significant difference in the overall AUC between the two groups (Figure S13B). Ex vivo biodistribution were also performed at 48 h and revealed a low level of lung radioactivity in rats injected with 3D hUC-MSCs.

In comparison to BALB/c mice, Wistar rats demonstrated a much earlier reduction in lung uptake. To delve deeper into the underlying reasons for this disparity, we compared the diameters of microvessels in the lungs from BALB/c mice and Wistar rats. As the diameters of lung capillaries were similar between mice (5 μm) and rat (6.6 μm) [28, 29], we focused our analysis on arterioles and venules surrounding alveoli. The immunofluorescence staining for α-SMA was conducted to identify microvessels. These microvessels are characterized by the presence of a lumen lined with α-SMA^+^ cells and filled with blood cells. As depicted in Figure S13C-S13E, the diameters of arterioles and venules in BALB/c mice were notably smaller than that in Wistar rats, measuring 50.93 ± 12.2 μm and 80.12 ± 25.85 μm, respectively.

## Discussion

In recent decades, MSCs have emerged as a promising therapeutic option for patients with limited or no alternative treatments due to their pleotropic therapeutic potential. Traditional MSC therapy primarily involves isolating MSCs from monolayers and administering them to recipients intravenously. However, the clinical application of intravenously injected MSCs is constrained by the tendency of most cells to accumulate in the lungs, thereby causing a swift decrease in survival rates and diminished therapeutic efficacy [20]. Sustained efforts have been dedicated to augmenting the therapeutic potential and homing efficiency of MSCs, including direct alteration (strategies such as genetic engineering and cell surface modification) and microenvironment regulation (strategies like spheroid culture and biomaterial usage) [30]. Regulating adhesive capacity and cell volume have garnered significant attention in this pursuit. However, their contribution in governing MSC homing and distribution remain incompletely understood. Capitalizing on the advantages of in vivo PET imaging, our study visualized the real-time distribution of MSCs to elucidate the effects of adhesion capacity and cell volume on cell homing, furnishing more evidences for optimizing clinical cell therapy.

Excess expression and activation of integrins in monolayer-derived MSCs were thought to be a critical cause of MSC entrapment in the lungs [31]. To reduce the adherence in the lung vasculature and improve the homing rate of MSCs, emerging molecules or peptides have been developed to functionally block adhesion receptors. As reported, polyethylenimine could bind integrin and CD106, thereby reducing MSC entrapment in the lungs [32]. Importantly, this surface modification did not compromise the viability, immunomodulation, and differentiation potential of MSCs. Also, ECM-derived peptides such as RGD and GFOGER have been utilized to functionalize hydrogels aimed at enhancing the survival and engraftment of MSCs in vivo by improving the expression of chemokines [30]. Our study applied adhesive peptides to temporarily block integrin or N-cadherin, minimizing the impact on the secretory profile of hUC-MSCs. We found that although these peptides can reduce cell adhesion by up to 43.5% in vitro without altering cell volume, the in vivo PET imaging revealed no significant reduction in MSC entrapment in the lungs. The inconsistency observed may be attributed to the differences between in vivo and in vitro circumstances. It is plausible that hUC-MSCs were physically intercepted within the lung vasculature, potentially obscuring the impact of adhesion capacity due to the influence of cell volume. Notably, the HAVDI pre-incubation group exhibited even higher lung retention, which may be associated with cell-cell interactions forming cell clusters which increase the cell volume. Besides, we found that all three peptides had no significant effect on the distribution of hUC-MSCs in the liver and spleen 48 h post-injection. The structure of the liver sinusoids and spleen sinusoids might intercept MSCs, potentially masking the influence of the cell surface peptide decoration. In addition, the binding of peptides to cell surface proteins is a reversible process, and the peptide may have dissociated from the cell by the time the cell reaches the liver and spleen.

The cell volume has long been recognized as a crucial factor contributing to lung entrapment during cell therapy due to the small diameter of lung capillaries, MSCs from various sources have been investigated to address this issue. Pre-treatment with a vasodilator has been shown to notably decrease lung entrapment and facilitate rapid lung clearance [33]. Recent studies demonstrated that culturing placenta-derived MSCs in a 3D environment or as spheroids for short duration can diminish cell volume by up to 40% and enhance their homing efficiency [34, 35]. Compare to bone marrow-derived MSCs in Wang’s work, our study indicated a faster lung clearance of hUC-MSCs, partially supporting the idea that bigger cells have higher lung retention. Consistently, Nystedt et al. demonstrated that the smaller diameter of hUC-MSCs may reduce lung entrapment, despite their higher expression levels of integrin α4 and integrin α6 compared to bone marrow-derived MSCs [9].

To delve deeper into the influence of two factors on MSC homing, we applied the hanging drop culture method to form MSC spheroids and track the distribution of disseminated cells in vivo. Culturing MSCs from various origins in a 3D environment exhibited improved homing efficiency through regulating cell volume and integrin expression levels [35, 36]. Consistent with previous research, the diameters of 3D-cultured hUC-MSCs significantly decreased by 34.6% without affecting their differential potential. But different from previous studies, the expression level of integrin subunits all increased except for integrin α6 after 3D culture in our study, which probably due to the different origin of MSCs and culture condition [37–39]. And the in vivo PET imaging revealed that 3D-cultured hUC-MSCs exhibited more rapid clearance from the lungs and more accelerated migration towards the livers and blood pool in comparison to 2D group. Based on these findings, we conferred that cell size regulates hUC-MSC in vivo distribution more effectively than adhesion capacity.

The variation in the diameter of the pulmonary vasculature among different species may have implications for the impact of cell size on lung entrapment. Specifically, the diameter of lung capillaries shows minimal differences among species, measuring approximately 5 μm in mice and monkey, and 5–10 μm in human [13, 40]. While larger vessels exhibit more noticeable differences in diameter between species. For instance, the diameter of arterioles and venules in mice and monkeys were approximately 38.26 μm and 92.89 μm, respectively. In line with these findings, our study determined that the diameters of arterioles and venules in BALB/c mice and Wistar rats were 50.93 ± 12.2 μm and 80.12 ± 25.85 μm, respectively. In humans, arterioles are defined as small vessels with diameters less than 100 μm [41]. Given that the diameter of hUC-MSCs is approximately 20 μm, it is likely that most intravenously injected cells would become trapped in precapillary vessels. And PET imaging revealed that 3D-cultured hUC-MSCs exhibited reduced lung entrapment in Wistar rats, despite their higher expression of integrin. Considering the comparable small vessel diameters between rats and humans, we inferred that cell size remains a predominant factor to be considered in clinical cell therapy.

Moreover, in our study using BALB/c mice, we observed that a decrease in lung uptake was often accompanied by a reduction in uptake within the liver and spleen. This observation aligns with findings reported by Patrick et al., who demonstrated that after intravenous injection, most MSCs detected in the liver were, in fact, non-viable cellular debris rather than intact living cells [42]. The likely explanation for this phenomenon is mechanical damage caused by shear stress during infusion, particularly given the narrow diameter of the pulmonary microvasculature in mice. Additionally, other physiological clearance mechanisms within the liver may further contribute to MSC apoptosis or degradation. Thus, while PET imaging provides valuable insights into the biodistribution of stem cells, assessing their viability in vivo requires complementary methods such as reporter gene imaging to distinguish between viable and non-viable cells. Nevertheless, PET imaging holds immense promise for advancing pharmacokinetic studies of innovative therapeutics, including cutting-edge therapies [43, 44]. By providing precise, real-time insights into the in vivo distribution and dynamic behavior of these complex agents, PET imaging has the potential to significantly enhance our understanding of their biodistribution, targeting efficiency, and therapeutic efficacy.

Within the landscape of regenerative medicine, the spatial distribution and successful migration of MSCs to target tissues are pivotal factors influencing their therapeutic efficacy. Upon intravenous infusion, more than 80% of MSCs are entrapped in lungs with less than 1% of cells reach ischemic heart and brain [45]. Thus optimizing in vivo biodistribution of MSCs is crucial to fully realize the therapeutic potential. Our results demonstrated that 3D culture effectively reduces MSC size while preserving pluripotency, which decreases lung entrapment and optimizes in vivo biodistribution. Also, 3D culture has been reported to improve anti-inflammatory properties, resistance to oxidative damage and large-scale expansion capacity [46–48], thereby providing new insights for the clinical translation of MSC therapy. However, several concerns need be addressed when using 3D cell cultures. It is crucial to carefully dissociate cells from 3D spheroids to maintain their viability and functional activity. To prevent cell damage, prolonged trypsin digestion should be avoided, and milder dissociation reagents like Accutase are recommended. Incomplete dissociation of 3D spheroids prior to in vivo administration can result in microvascular embolization, underscoring the importance of thorough cell separation and purification. When necessary, techniques such as gradient centrifugation can be employed to ensure a pure population of single, dissociated cells. Moreover, the adoption of alternative 3D culture platforms, such as hydrogels, may yield MSCs with enhanced quality and therapeutic potential [49].

Furthermore, scalability is a crucial factor when considering the practical application of 3D culture methods for producing cell therapies at a clinical scale. Although 2D cultures are more widely used in bioreactor systems and are straightforward to scale up, they have shown the functional defects and lack of maturity by which the conditions supplied are different from the three-dimensional originals [50]. Thus, 3D suspension culture systems for expansion and differentiation bring hope for cell therapy [51]. Many attempts have been made to establish a robust and economical stem cell suspension culture system, including dynamic stirring method, microcarriers or microcapsules carrying cells, cell aggregates, and ultra-low attachment materials [52, 53]. And a yield of 1.5 × 10^9^ cells per 1.5 L could be reached while maintaining normal characteristics of stem cells. Therefore under the optimal experimental protocol, the 3D culture system offering a promising avenue for mass production and clinical and applications of cell therapy.

## Conclusion

In this study, we utilized PET to visualized the real-time biodistribution of transplanted cells in vivo. By employing this pioneering imaging technique, we elucidated that cell size serves as the primary biophysical determinant governing the localization of hUC-MSCs within host tissues. Our findings provide insights to guide the design of hUC-MSC-based therapies, with the aim of optimizing their therapeutic efficacy for tissue repair and regeneration.

## Electronic supplementary material

Below is the link to the electronic supplementary material.

Supplementary Material 1

## Data Availability

All data generated or analyzed during this study are included in this published article and its supplementary information files.

## Abbreviations

α-SMA: α-smooth muscle actin
ARS: Alizarin red S
AUC: area under the curve
BSA: bovine serum albumin
CI: collagen I
DAPI: 4,6-diamidino-2-phenylin-dole
DMSO: dimethyl sulphoxide
FN: fibronectin
FITC: fluorescein 5-isothiocyanate
HE: hematoxylin and eosin
HEPES: 4-(2-hydroxyethyl)-1-piperazineethanesulfonic acid
MIP: maximum intensity projection
MSC: mesenchymal stem cell
PET: positron emission tomography
hUC-MSC: human umbilical cord blood-derived MSC
3D: three dimensional
%ID/g: percentage injected dose per gram
%ID/ml: percentage injected dose per milliliter

## Declarations

### Artificial intelligence

The authors declare that artificial intelligence was not used in this study.
