# Supplementary material for: Impact of mesenchymal stem cell size and adhesion modulation on in vivo distribution: insights from quantitative PET imaging
Source: Stem Cell Res Ther. 2024 Nov 28;15:456. doi: 10.1186/s13287-024-04078-4 (PMC11606219; doi:10.1186/s13287-024-04078-4)
Supplement: Supplementary file 1 — Supplementary Material 1 [file 13287_2024_4078_MOESM1_ESM.docx]

**Supporting Information**

**Impact of Mesenchymal Stem Cell Size and Adhesion Modulation on In Vivo Distribution: Insights from Quantitative PET Imaging**

Xin Ji^#1^, Lizhen Wang^#2,3^, Yudan Zhong^2,3^, Qian Xu^2,3^, Junjie Yan^2,3^, Donghui Pan^3^, Yuping Xu^2,3^, Chongyang Chen^3^, Jing Wang^4^, Guangji Wang^5^, Min Yang^2,3^, Tiannv Li^1^, Lijun Tang^1^*, Xinyu Wang^2,3^*

1 Department of Nuclear Medicine, the First Affiliated Hospital of Nanjing Medical University, Jiangsu Province Hospital, Nanjing 210029, P.R. China

2 Department of Radiopharmaceuticals, School of Pharmacy, Nanjing Medical University, Nanjing 211166, P.R. China

3 NHC Key Laboratory of Nuclear Medicine, Jiangsu Key Laboratory of Molecular Nuclear Medicine, Jiangsu Institute of Nuclear Medicine, Wuxi 214063, P.R. China

4 Jiangsu Renocell Biotech Co., Ltd., Nanjing 211100, P.R. China

5 Key Laboratory of Drug Metabolism and Pharmacokinetics, State Key Laboratory of Natural Medicines, China Pharmaceutical University, Nanjing 211198, P.R. China


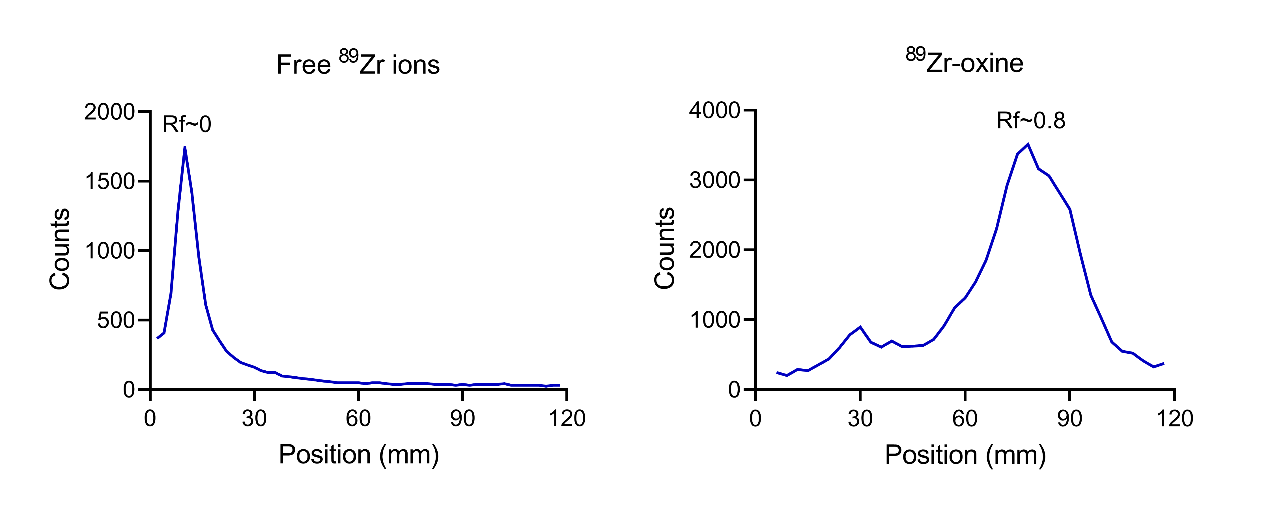


Figure S1. Radio-TLC analysis of free ^89^Zr^4+^ and ^89^Zr-oxine. The mobile phase is 100% methanol.


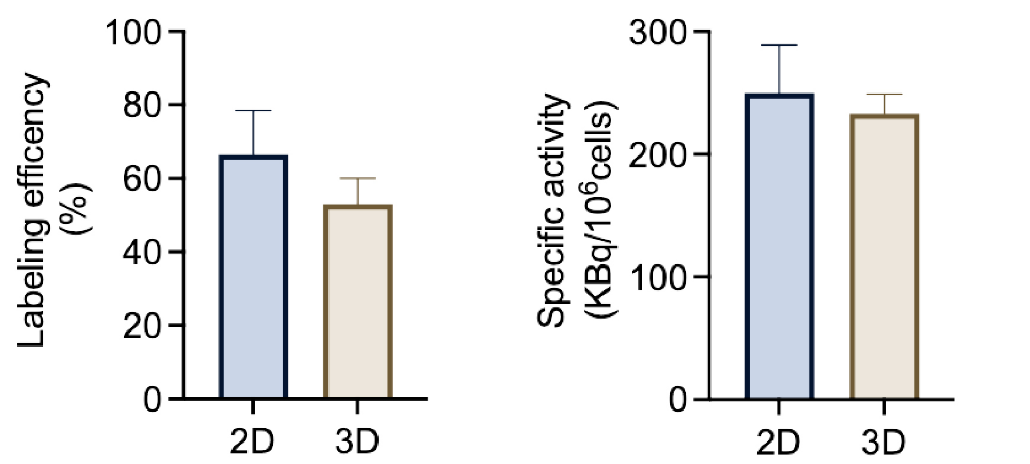


Figure S2. ^89^Zr labelling efficiency of 2D and 3D cultured MSCs and specific activity (KBq/10^6^ cells) of ^89^Zr labelled 2D and 3D cultured hUC-MSCs.


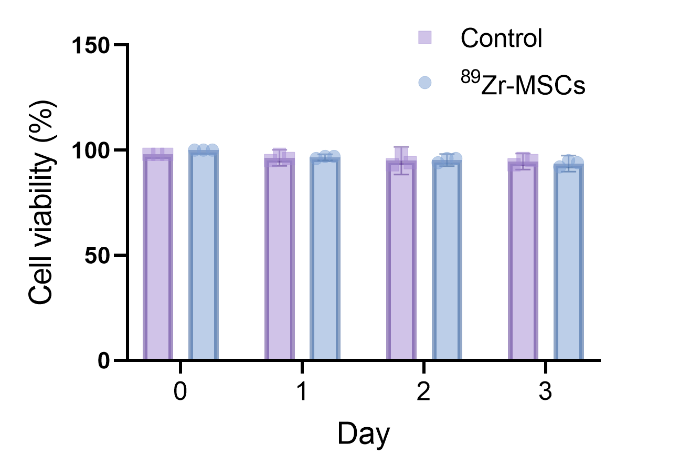


Figure S3. The viability of ^89^Zr-labeled hUC-MSCs was assessed over three days and compared to that of untreated control hUC-MSCs.


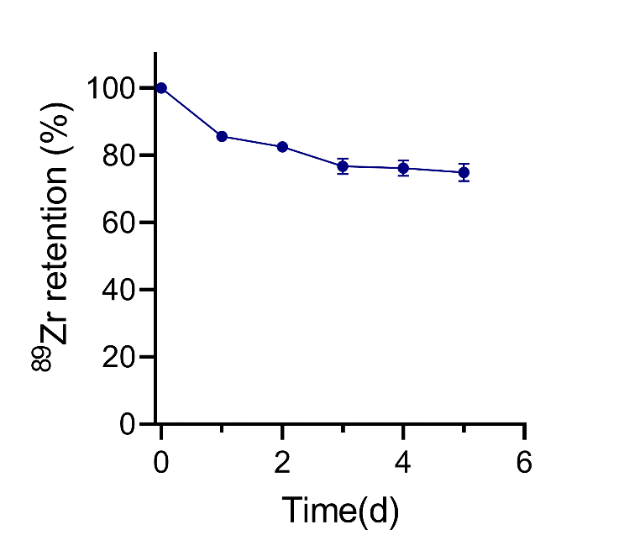


Figure S4. ^89^Zr retention in radiolabeled hUC-MSCs over 5 days following initial radiolabeling.


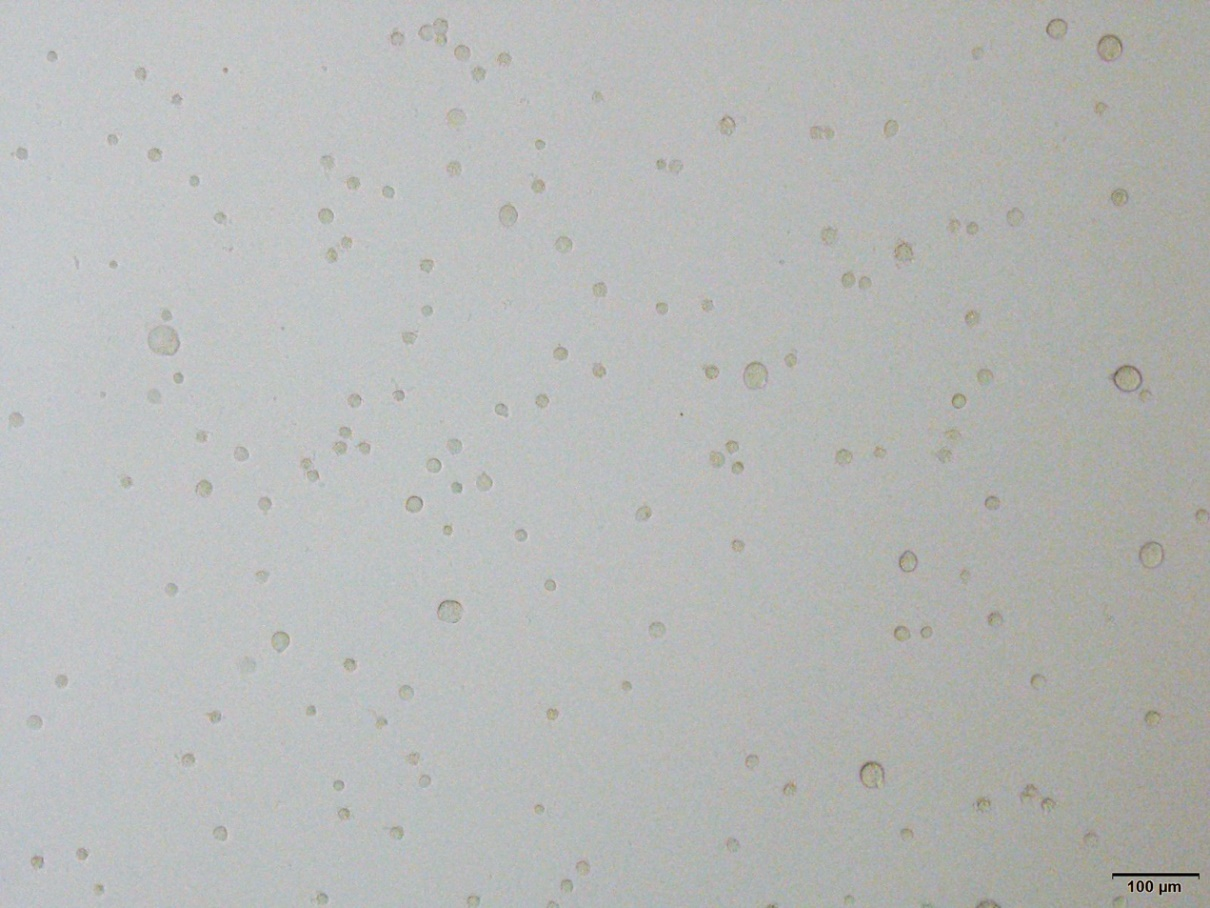


Figure S5. High-resolution images of hUC-MSC cultures in 2D immediately following cell passage.


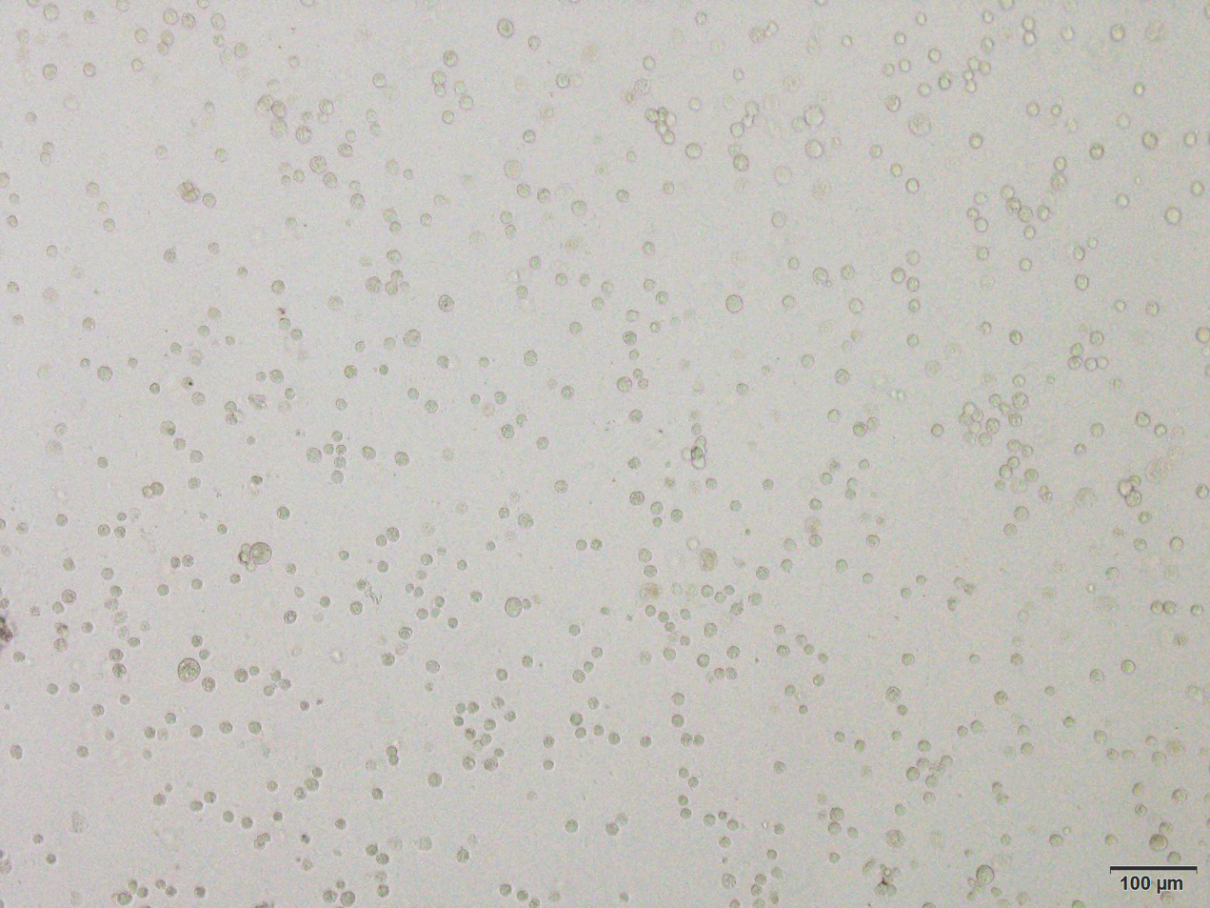


Figure S6. The higher resolution figures of hUC-MSCs cultures in 3D for 24 h.


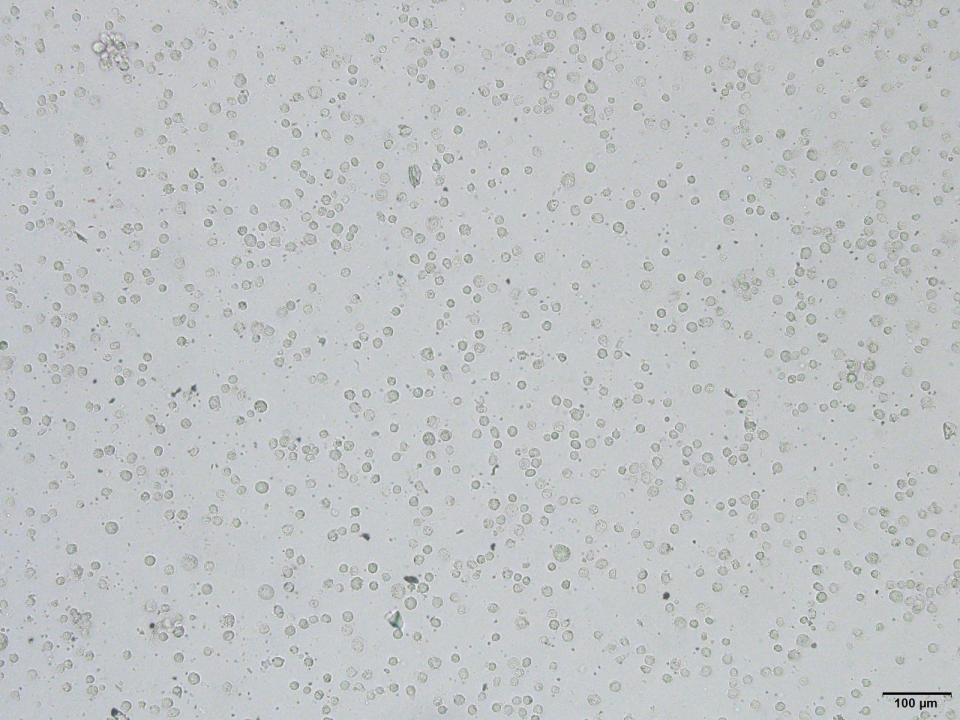


Figure S7. The higher resolution figures of hUC-MSCs cultures in 3D for 48h.


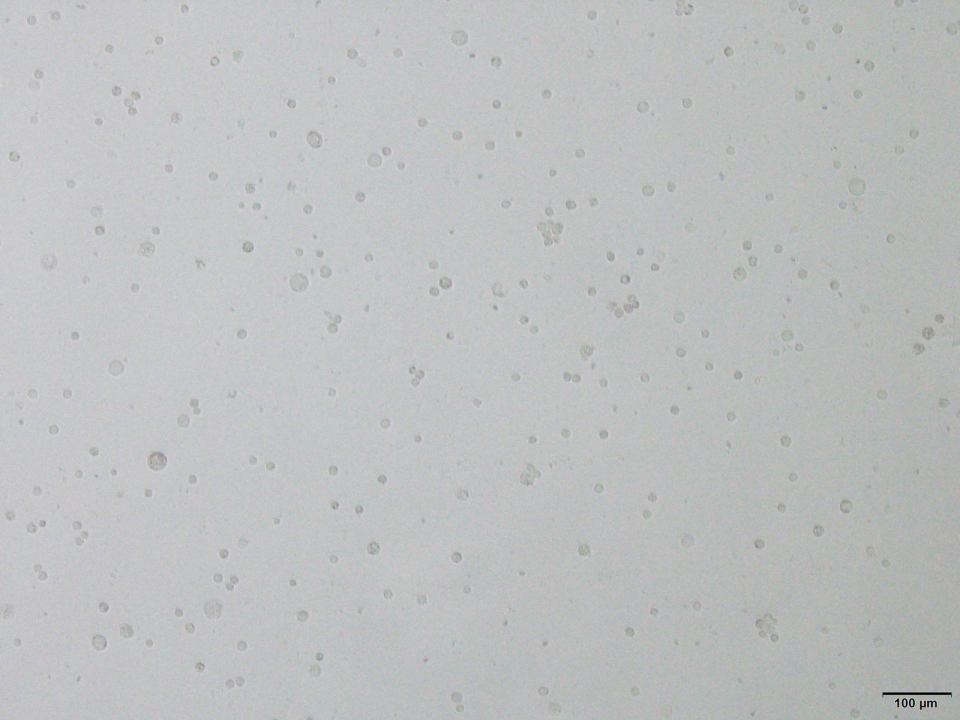


Figure S8. The higher resolution figures of hUC-MSCs cultures in 3D for 72 h.


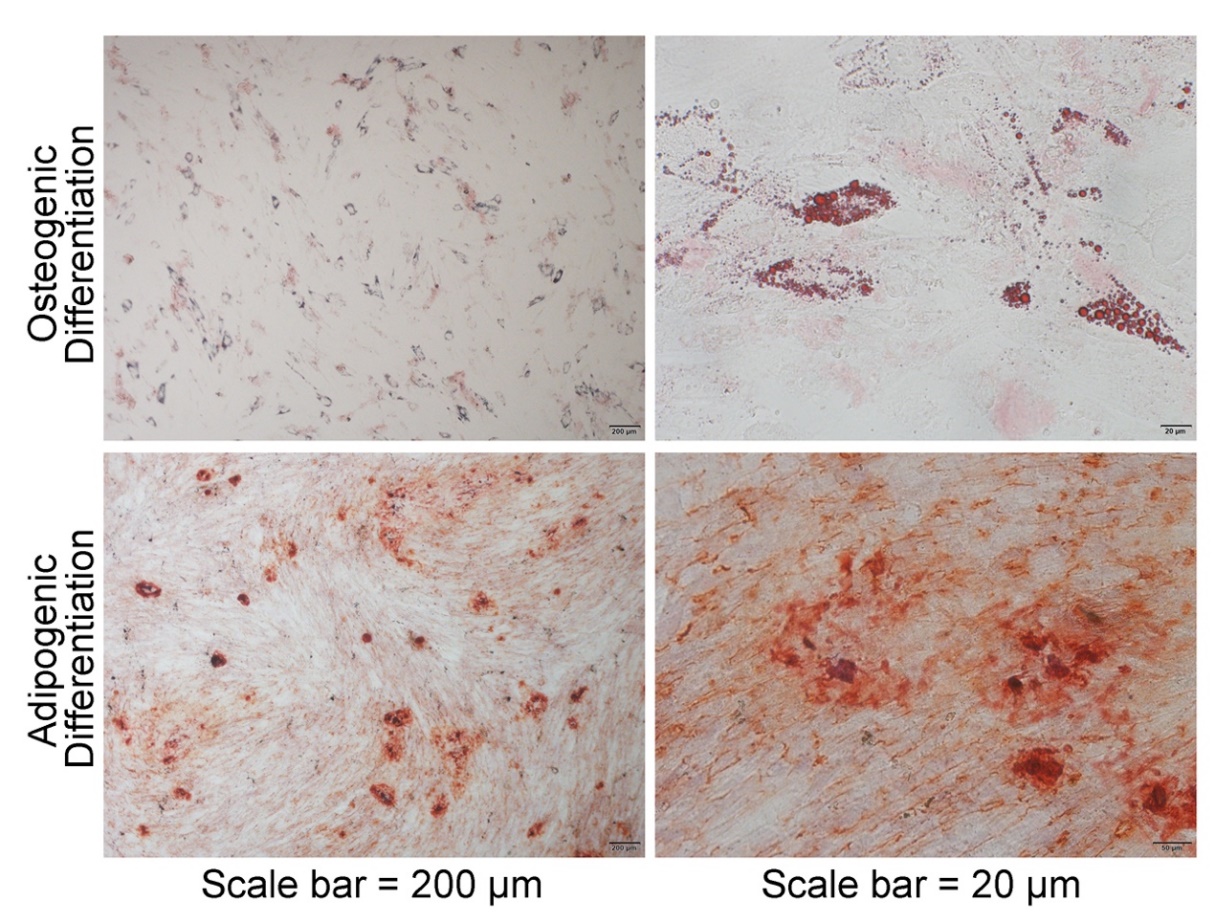


Figure S9. The higher resolution figures of Alizarin red staining and Oil Red O staining of 2D cultured hUC-MSCs.


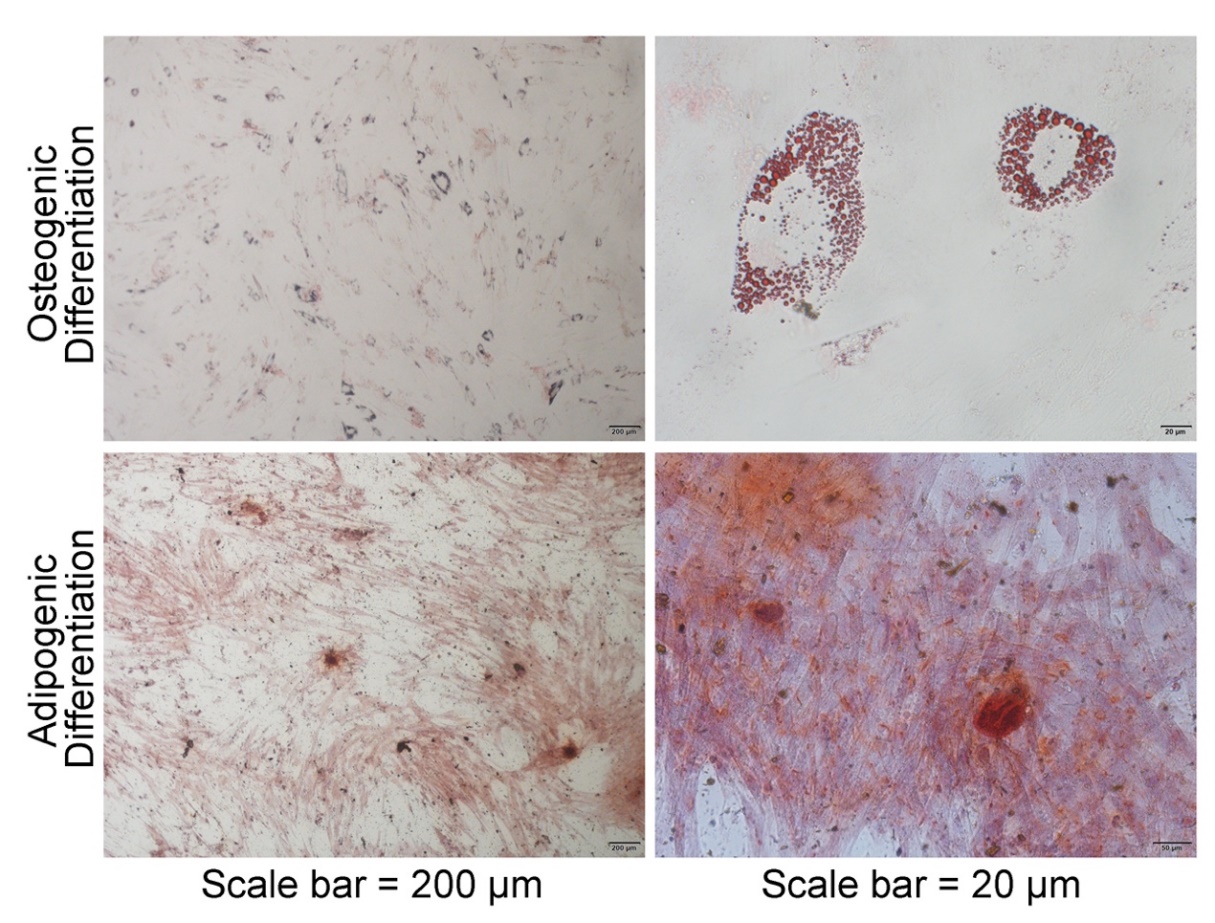


Figure S10. The higher resolution figures of Alizarin red staining and Oil Red O staining of 3D cultured hUC-MSCs.


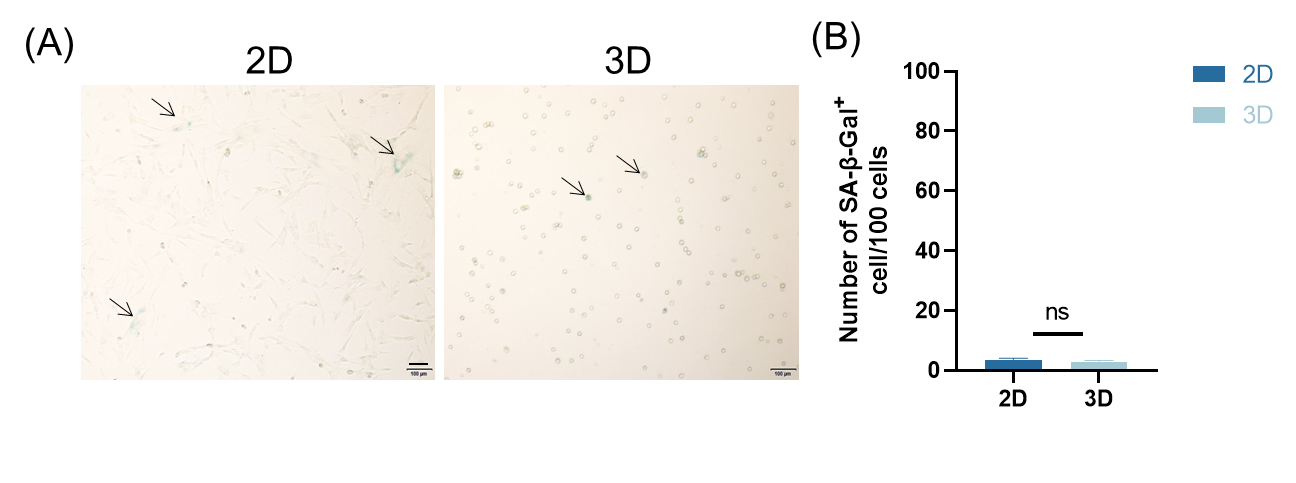


Figure S11. SA-β-Gal staining of MSCs. (A) Representative images showing the SA-β-Gal staining results of MSCs cultured under 2D and 3D conditions, respectively. (B) Quantitative analysis of the percentage of senescent MSCs in each group. Data are presented as means ± SD (n = 100). ns indicates no significant difference.


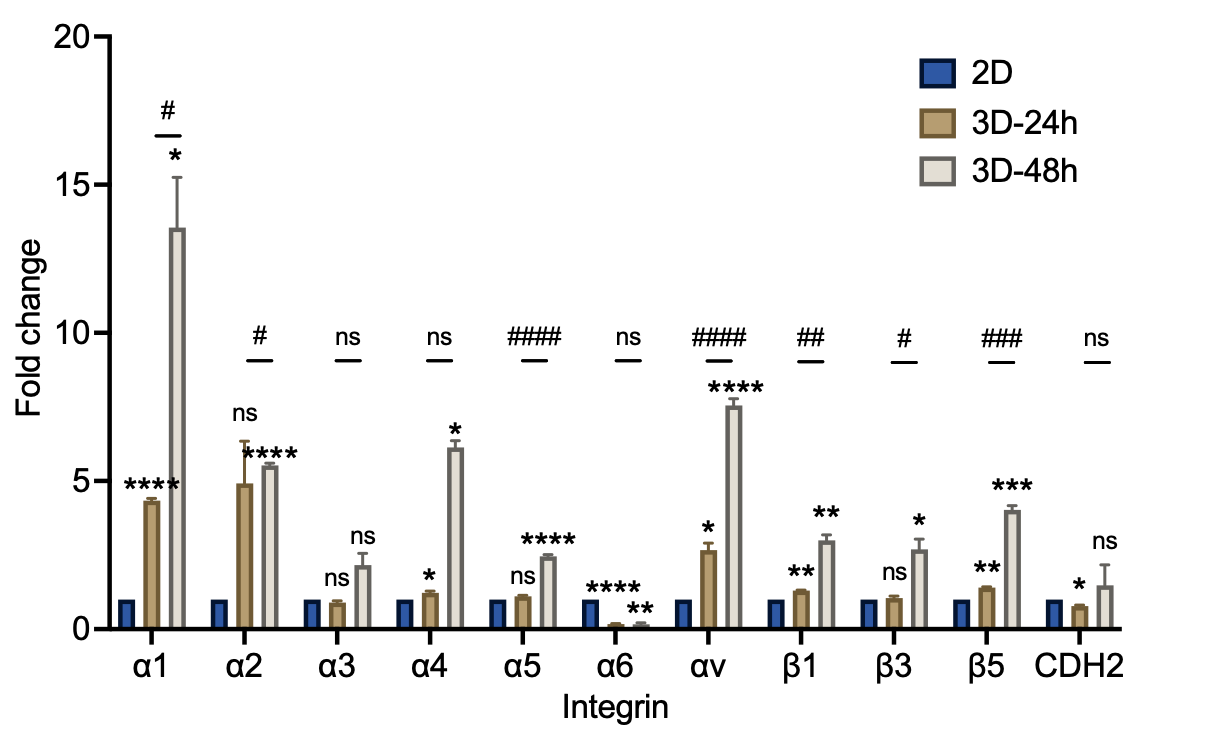


Figure S12. Expression of integrin and N-cadherin (CDH2) after 3D cultivation. The expression level of cell surface receptors after 0 h (2D), 24 h (3D-24h), and 48 h (3D-48h) of 3D cultivation were further quantified using RT-PCR (n=3). Values are expressed as the means ± SD. *, represents a comparison to 2D. *, p < 0.05; **, p < 0.01; ***, p < 0.001; ****, p < 0.0001. #, represents a comparison to 3D-24h. #, p < 0.05; ##, p < 0.01; ###, p < 0.001; ####, p < 0.0001; ns, not significant, p > 0.05.


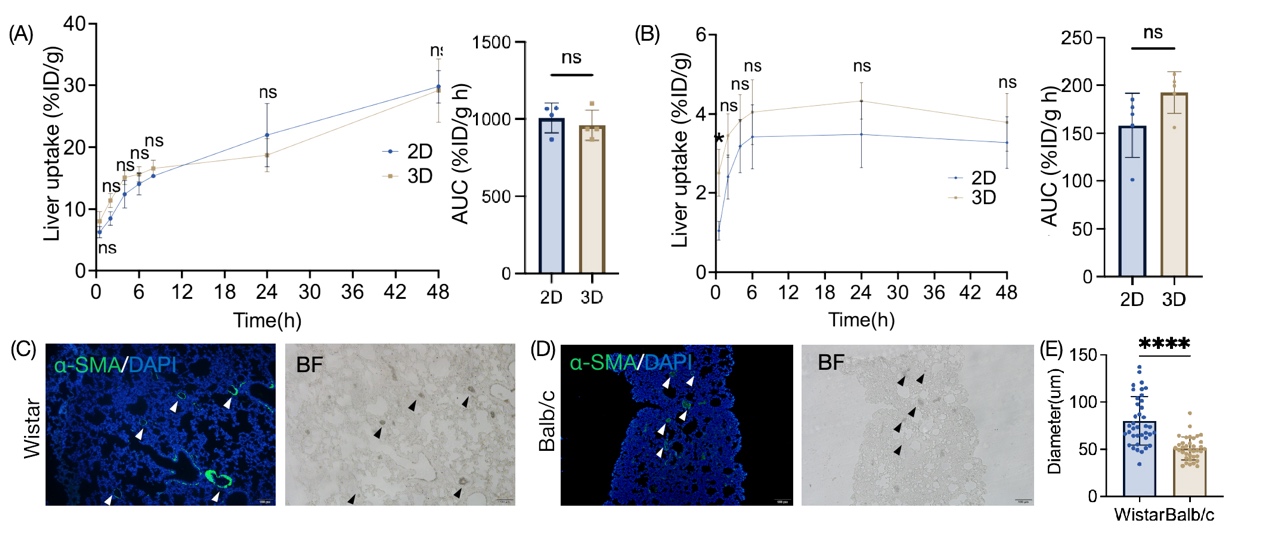


Figure S13. Various liver distribution and vasculature diameter between BALB/c mice and Wistar rats. Liver uptake in BALB/c mice (A) and Wistar rats (B), (n=4 for Balb/c and n=5 for Wistar). The immunofluorescent staining of α-SMA (green) and nuclei (blue) of lung microvessels from Wistar rats (C) and Balb/c mice (D). Arterioles or venules containing blood cells in their cavity are indicated with triangles. Bright-field (BF) images are shown together. Scale bar = 100 μm. Diameters of mocrovessels are also displayed in the bar chart (E). Values are expressed as the means ± SD. *, p < 0.05; **, p < 0.01; ***, p < 0.001; ****, p < 0.0001; ns, not significant, p > 0.05.


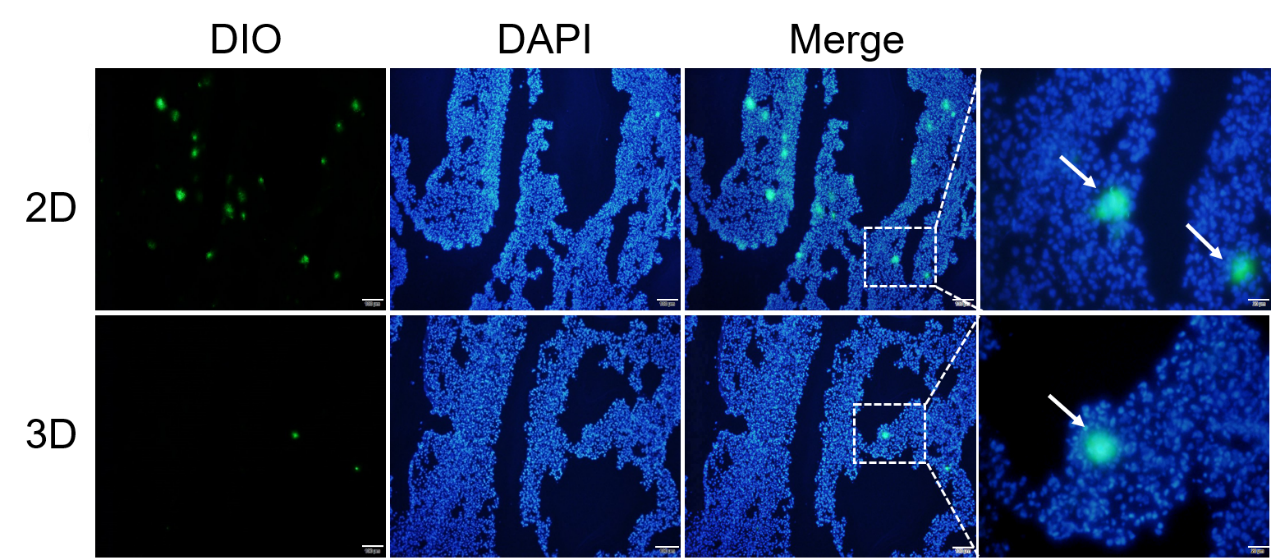


Figure S14. Fluorescently stained tissue sections illustrating the distribution of 2D and 3D cultured hUC-MSCs within the lungs of BALB/c mice. Prior to injection, hUC-MSCs were labeled with DiO (green fluorescence), while tissue nuclei were counterstained with DAPI (blue fluorescence), enabling clear visualization of cell localization in lung tissue. Scale bar is 100 μm.


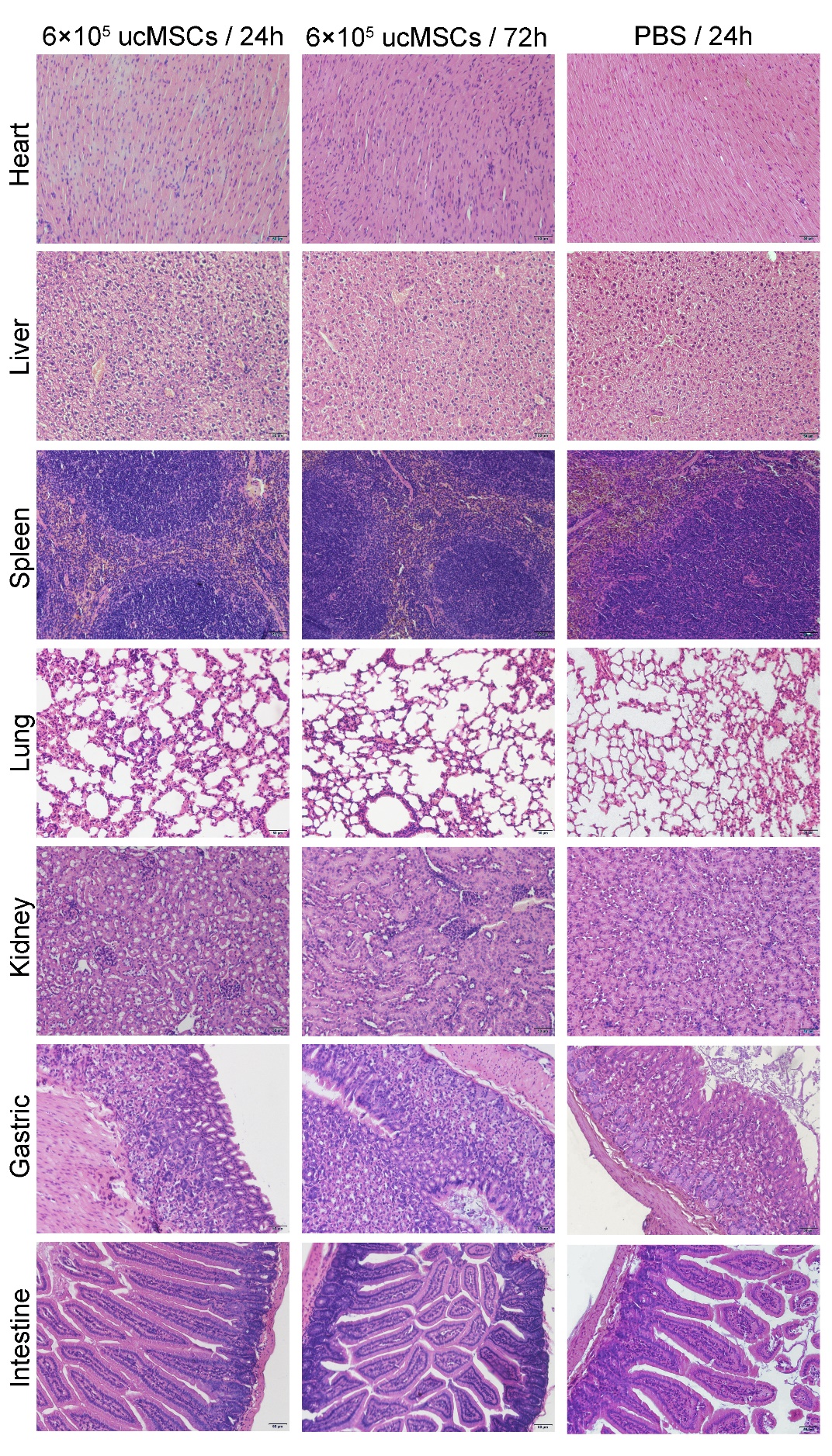


Figure S15. Hematoxylin and eosin-stained tissue sections from BALB/c mice treated with 6×10⁵ hUC-MSCs for 24 hours and 72 hours, compared to PBS control. These images provide a comparative histological overview of tissue morphology and cellular response following hUC-MSCs administration.

Table S1. Primer sequences used for PCR.

|  | Forward primer | Reverse primer |
| --- | --- | --- |
| integrin α1 | GGTTCCTACTTTGGCAGTATT | AACCTTGTCTGATTGAGAGCA |
| integrin α2 | GGAACGGGACTTTCGCAT | GGTACTTCGGCTTTCTCATCA |
| integrin α3 | AAGGGACCTTCAGGTGCA | TGTAGCCGGTGATTTACCAT |
| Integrin α4 | GCTTCTCAGATCTGCTCGTG | GTCACTTCCAACGAGGTTTG |
| integrin α5 | TGCAGTGTGAGGCTGTGTACA | GTGGCCACCTGACGCTCT |
| integrin α6 | TTGAATATACTGCTAACCCCG | TCGAAACTGAACTCTTGAGGATAG |
| integrin αV | AATCTTCCAATTGAGGATATCAC | AAAACAGCCAGTAGCAACAAT |
| integrin β1 | GAAGGGTTGCCCTCCAGA | GCTTGAGCTTCTCTGCTGTT |
| integrin β3 | CCGTGACGAGATTGAGTCA | AGGATGGACTTTCCACTAGAA |
| integrin β5 | GGAGCCAGAGTGTGGAAACA | GAAACTTTGCAAACTCCCTC |
| N-cadherin (CDH2) | CCTCCAGAGTTTACTGCCATGAC | GTAGGATCTCCGCCACTGATTC |
| GAPDH | GCACCGTCAAGGCTGAGAAC | TGGTGAAGACGCCAGTGGA |
